# Procedural Sedation Using a Propofol–Ketamine Combination (Ketofol) vs. Propofol Alone in the Loop Electrosurgical Excision Procedure (LEEP): A Randomized Controlled Trial

**DOI:** 10.3390/jcm8070943

**Published:** 2019-06-28

**Authors:** Chahyun Oh, Yeojung Kim, Hongsik Eom, Sookyoung Youn, Sangmin Lee, Young-Bok Ko, Heon Jong Yoo, Woosuk Chung, ChaeSeong Lim, Boohwi Hong

**Affiliations:** 1 Department of Anesthesiology and Pain Medicine, College of Medicine, Chungnam National University, 266 Munhwa-ro, Jung-gu, Daejeon 35015, Korea; 2 Department of Anesthesiology and Pain Medicine, Chungnam National University Hospital, 282 Munhwa-ro, Jung-gu, Daejeon 35015, Korea; 3 Department of Obstetrics and Gynecology, Chungnam National University Hospital, 282 Munhwa-ro, Jung-gu, Daejeon 35015, Korea

**Keywords:** Sedation, propofol, ketamine, conization, drug combinations

## Abstract

Background: Although the loop electrosurgical excision procedure (LEEP) is a brief procedure, it can cause severe pain and discomfort to patients in the absence of adequate sedation. An admixture of ketamine with propofol (ketofol), may reduce patient movement due to insufficient sedation while providing hemodynamic and respiratory stability. This study evaluated the ability of two ratios of a propofol–ketamine combination, compared with propofol alone, to reduce patient movement during procedural sedation for LEEPs. Methods: One hundred and twenty women scheduled for a LEEP were randomly assigned to three groups. Anesthesia was induced with 1 mg/kg propofol (group P), 1 mg/kg propofol and 0.33 mg/kg ketamine (group K1), or 1 mg/kg propofol and 0.66 mg/kg ketamine (group K2). The primary outcome was the incidence of adduction motion in the lower extremities during the procedure. The requirements for respiratory interventions, changes in vital signs, sedation score, additional anesthetic usage, and surgeon and patient satisfaction were also evaluated. Results: The incidence of adduction motion was significantly lower in groups K1 and K2 than in group P (overall *p*-value <0.001) but did not differ significantly in groups K1 and K2. Group K2 needed more jaw thrust maneuvers than group K1. Additional propofol usage was lower and surgeon satisfaction scores higher in groups K1 and K2 than in group P. Conclusion: A propofol–ketamine combination is more effective than propofol alone in reducing procedural interference during LEEPs. However, increasing the dose of ketamine showed no additional benefit.

## 1. Introduction

Procedural sedation has been used in various medical procedures worldwide. This sedation should not only calm the patient and/or provide analgesia but should take into account various factors, such as preexisting medical conditions and potential risks of hemodynamic and respiratory compromise [1]. 

Propofol is a hypnotic agent, which has a rapid onset and offset of drug effect. Due to its extensive initial redistribution, it allows brief hypnosis, which is suitable for simple outpatient procedures. It also has other desirable clinical features such as amnesia, antiemesis, and is almost free of drug hangover. However, propofol does not have analgesic properties and may dose-dependently increase the risks of hemodynamic and respiratory compromise [2].

Ketamine is quite different from propofol. It produces a distinct form of anesthesia, which is called “dissociative anesthesia”, and also provides analgesia. It has a sympathomimetic effect and preserves spontaneous ventilation, which is desirable for procedural sedation [2]. However, it can cause psychomimetic side effects such as vivid dreams and emergence agitation [3,4].

The combination of ketamine and propofol (ketofol) is theoretically expected to have the advantages of both drugs and to complement each other’s disadvantages. Hemodynamic compromise induced by propofol may be compensated by the sympathomimetic effect of ketamine. Psychomimetic adverse effects are known to be reduced by concomitant use of propofol [2]. Indeed, the combination has been shown to be useful in many clinical situations, with better profiles in hemodynamic stability, respiratory depression, analgesia, and recovery than each agent alone [2,5,6,7,8,9,10,11,12,13].

The loop electrosurgical excision procedure (LEEP) is a major treatment and diagnostic procedure commonly performed in an outpatient setting. Despite the fact that a LEEP is a brief procedure, it can cause significant pain and discomfort to the patient. On average, a visual analogue score (VAS) of 4 points was reported due to electrical excision during the procedure even with local anesthesia [14,15]. The cervix is a small procedural target and located deep inside the vagina. It is difficult for the surgeon to perform the procedure accurately if the patient moves during the procedure. Therefore, inadequate sedation can cause great discomfort, not only to the patient but to the surgeon, and may result in various complications. No study to date has evaluated the usefulness of sedation using a propofol–ketamine combination in women undergoing LEEPs. This study, therefore, evaluated the effectiveness of a propofol–ketamine combination in LEEPs by comparing the incidence of procedural interference in patients receiving sedation with a propofol–ketamine combination, administered at 1:3 and 2:3 ratios of ketamine:propofol, or propofol alone. We hypothesized that a propofol–ketamine combination would reduce procedural interference during LEEPs.

## 2. Materials and Methods

### 2.1. Study Design and Participants

This randomized controlled, double-blinded, parallel group, three-arm, superiority trial was approved by a tertiary care hospital institutional review board of South Korea (2018-04-016) and registered with the Clinical Research Information Service, a clinical trial registry in South Korea (KCT0003208). The trial cohort consisted of women aged 20–70 years with American Society of Anesthesiologists (ASA) physical status I or II scheduled for a LEEP at the tertiary care hospital from May 2018 to February 2019. Patients were excluded if they refused to participate in the study or had any known contraindications to either ketamine or propofol. All participants provided written informed consent on the day before the procedure.

### 2.2. Randomization and Minimization of Bias

Patients were randomly assigned to undergo procedural sedation with propofol or a propofol–ketamine combination using a table of random numbers. Block randomization was performed on a website (www.randomization.com) with a random sequence generator, a random block size of 3, 6 and a 1:1:1 allocation. To conceal the allocation, the sequence was uploaded by REDCap (Research Electronic Data Capture) software in our hospital and was accessible only to the researcher who prepared the study drug. The allocation was revealed after the participant entered the operating room, with the study drug prepared according to the assignment. The researcher was not involved in patient monitoring or outcome analysis. Standard solutions consisted of 50 mg/mL ketamine and 10 mg/mL propofol. For blinding, transparent syringes containing 1 mL of 0.33 mg/kg or 0.66 mg/kg ketamine in normal saline, or saline alone, were prepared, with their contents added to 10 mL syringes containing 1 mg/kg propofol. Except for the researcher who prepared the syringes, other individuals who participated in the procedure, including the anesthesiologist, surgeon, nurse, and patient, were blinded to drug the assignment. 

### 2.3. Anesthetic Procedures

Then, 0.2 mg glycopyrrolate was injected intramuscularly 30 minutes before the patient entered the operating room. Three lead electrocardiography, pulse oximetry, and a noninvasive intermittent blood pressure cuff were used to monitor SpO2, heart rate, and systolic, diastolic, and mean blood pressure. Oxygen supplementation, at a rate of 5 L/min, was initiated via a simple facial mask prior to sedation. Sedation was induced by bolus intravenous injection of 1 mg/kg propofol (group P), 1 mg/kg propofol and 0.33 mg/kg ketamine (group K1), or 1 mg/kg propofol and 0.66 mg/kg ketamine (group K2). The target level of sedation was set at 6 on the Ramsay sedation scale (RSS). If the target level of sedation was not achieved, an additional 20 mg propofol was injected and repeated if necessary, to a maximum 40 mg additional propofol. If the target level of sedation was still not achieved, sevoflurane inhalation was performed at the attending anesthesiologist’s discretion. The procedure was started after the patient achieved the target depth of sedation. Vital signs, including blood pressure, heart rate, SpO2 and depth of sedation, were assessed immediately before the injection of study drug (baseline), 30 s after injection, and every 2 minutes until the end of the procedure. End-tidal carbon dioxide (EtCO2) was monitored continuously by a sidestream sampling line inserted into the facial mask. Jaw thrust maneuvers were initially performed when apnea occurred, as determined by capnography. If effective ventilation did not occur after the initial response, bag-mask ventilation was performed. At the end of the procedure, patients were transferred to the post-anesthesia care unit after confirming the presence of spontaneous breathing and the ability to follow a simple verbal command and monitored until they met discharge criteria (modified Aldrete score > 8). All measurements and outcomes were recorded by the attending anesthesiologist. Study data were collected and managed using REDCap electronic data capture tools.

### 2.4. Outcome Assessments

The primary outcome was the incidence of adduction motions in the lower extremities, which indicate procedural interference due to inadequate sedation or analgesia during the procedure. Adduction motion was defined as a clinically observable active movement of the lower extremity (not necessarily confined to pure adduction of the thigh) caused by procedural stimulation. Secondary outcomes included changes in vital signs, sedation scores, requirements for jaw thrust maneuvers or bag-mask ventilation, requirements for additional propofol or sevoflurane, the memory of intraoperative pain, agitation during recovery, total duration of the procedure, length of stay in the post-anesthesia care unit, and surgeon and patient satisfaction scores. The memory of intraoperative pain was queried when the patient is fully awake at the post-anesthesia care unit. The occurrence of recovery agitation was determined by the need for physical restraint. The satisfaction score was measured on 5-point Likert scales.

### 2.5. Statistical Analysis

The required sample size was based on a pilot study and calculated using G*Power (version 3.1, Franz Faul and Edgar Erdfelder, Trier, Germany). The pilot study found that adduction motion was observed in 90% (9/10) of patients in group P and 50% (5/10) patients in group K1. Based on a 40% absolute between-group difference in the incidence of adduction motions, 34 subjects in each group would need to have a power of 90% and a risk of 1.6% (1/3 of 5) for type I errors. Based on an estimated dropout rate of 15%, 120 subjects were recruited. 

All analyses were performed in the intention to treat (ITT) population. Continuous variables were analyzed by the analysis of variance (mean ± standard deviation) or the Kruskal–Wallis H test (median [interquartile range]) depending on the results of Shapiro–Wilk tests of normality. Categorical variables were analyzed using χ2 or Fisher’s exact test and recorded as number (%). A two-tailed *p*-value < 0.05 was considered statistically significant. Pairwise comparisons were analyzed using the Mann–Whitney test for continuous variables and χ2 or Fisher’s exact test for categorical variables, with *p*-values < 0.017, using Bonferroni’s correction, considered statistically significant. To account for missing values and correlations between multiple measurements, a linear mixed-effect model was used to analyze vital signs and sedation score. Group, time, and group-time interaction were considered as fixed factors, and an individual was considered as a random factor. Data were analyzed using R software version 3.5.2 (R Project for Statistical Computing, Vienna, Austria). 

## 3. Results

**Figure 1:**
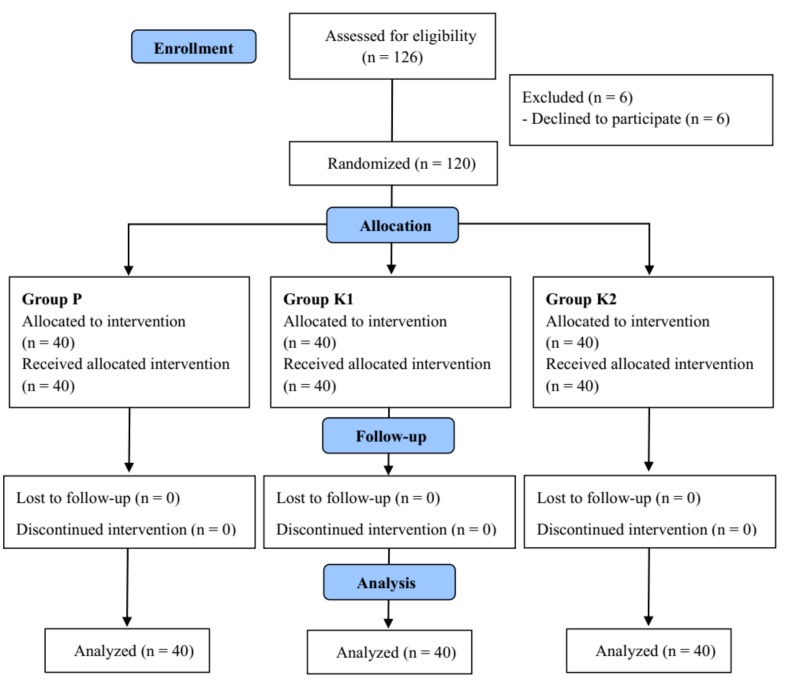
Consolidated Standards of Reporting Trials (CONSORT) flow chart.

Of the 126 patients assessed for eligibility, six refused to participate and were excluded. Of the 120 patients randomized, none was excluded from the analysis. The Consolidated Standards of Reporting Trials (CONSORT) diagram is shown in Figure 1, and the characteristics of the patients in the three groups are illustrated in Table 1.

**Figure 2:**
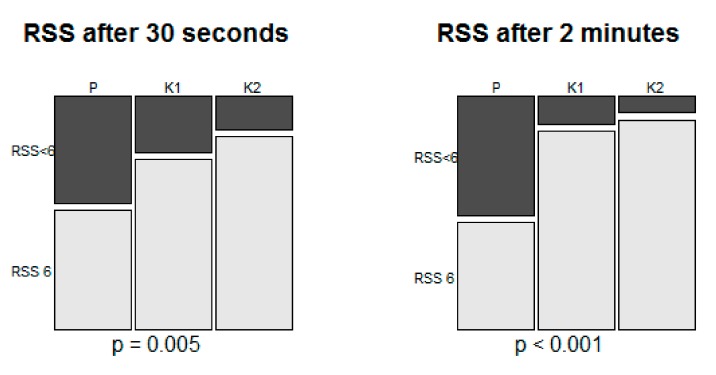
The proportions of the patients who reached six on the RSS at 30 s and 2 min after induction. RSS, Ramsay sedation scale.

Of the 120 patients, 85 (70.8%) reached six on the RSS within 30 s and then this number increased to 91 (75.8%) at 2 min after induction. The proportions of the patient who reached six on the RSS at these time points showed significant differences among groups (Figure 2).

The primary outcome of the study, the incidence of adduction motion of the lower extremities, differed significantly among the three groups, being 10.0% in group K2, 32.5% in group K1 and 67.5% in group P (overall *p* value <0.001) (Table 2). Compared with the patients in group P, those in groups K1 (*p* = 0.004; relative risk [RR] = 2.08; 95% confidence interval [CI] = 1.27–3.41) and K2 (*p* < 0.001; RR = 6.75; 95% CI = 2.60–17.53) had a lower incidence of adduction motion. However, the difference between groups K1 and K2 was not significant (*p* = 0.029).

**Figure 3:**
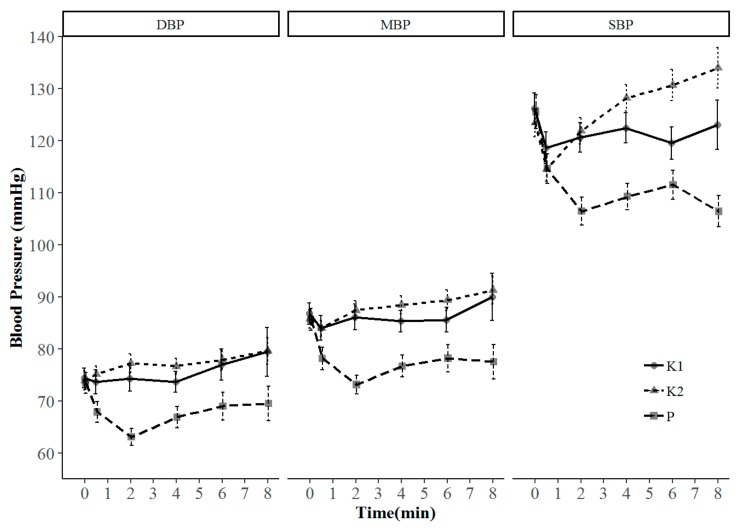
Blood pressure during the procedure. Data points represent the mean value (error bar: 95% confidence interval) measured at each time point. DBP, diastolic blood pressure; MBP, mean blood pressure; SBP, systolic blood pressure.

Secondary outcomes are shown in Table 3. The requirement for jaw thrust maneuvers was higher in group K2 than in group K1, although the requirement for bag-mask ventilation did not differ among the three groups. Additional propofol use was lower, and surgeon satisfaction scores were higher in groups K1 and K2 than in group P, but there were no differences between groups K1 and K2. There was no significant difference in the occurrence of recovery agitation among groups. No patient exhibited significant hypotension. Although systolic blood pressure tended to decrease in groups K1 and P, it remained significantly more stable in group K2 (*p* < 0.001) (Figure 3). Other vital signs and sedation scores did not differ significantly among the three groups throughout the procedure. 

## 4. Discussion

The current study demonstrated that the addition of ketamine to propofol can reduce procedural interference due to inadequate sedation. Although the LEEP is a relatively brief procedure, it is performed with the patient in a lithotomy position, with the procedural field being narrow and not readily accessible. Although it is short-lived in most patients, there is considerable pain during the procedure if local anesthetics are omitted [16]. Even in cases with local anesthetics, anesthesia is not always successful and the evidence of its efficacy in LEEPs is not solid [17]. Thus, adequate sedation during a LEEP is beneficial for both patients and physicians. 

In our study, adduction motion occurred in two-thirds of patients in group P and one-third of patients in group K1. Similar results were reported in several previous studies. For example, a comparison of sedation quality during placement of retrobulbar nerve block found that 61% of patients sedated with propofol and 36% of patients sedated with a 3:1 ratio of propofol:ketamine grimaced or moved their extremities during needle insertion [18]. A study investigating the dose-dependent effects of ketamine during propofol sedation in women undergoing breast biopsy found that 44% of patients sedated with propofol alone required rescue analgesic due to pain or discomfort from local anesthetic infiltration, with significantly fewer patients sedated with a propofol–ketamine combination requiring rescue analgesic [19]. A comparison of propofol and 1:1 and 2:1 ratios of propofol:ketamine in men undergoing transrectal ultrasound-guided prostate biopsy found that rates of patient movement during the procedure were much lower in the combination groups [20]. Despite differences in demographic characteristics, the use of premedication, procedural pain, and sedative dosages and injection rates among these studies, a propofol–ketamine combination provided better procedural conditions than propofol, without undermining respiratory and hemodynamic stability. Similar results favoring the combination were reported in emergency department (ED) settings, with rates of procedural agitation or interference being much lower than in previous studies [21,22]. Pre-procedural analgesia and differences in the type of procedure may affect these rates.

Respiratory stability is the core safety issue associated with sedation using a propofol–ketamine combination [8,11,21,22,23,24]. Several case series and randomized control trials performed before 2012 provided equivocal evidence for the benefits of a propofol–ketamine combination [25]. Three consecutive well designed randomized control trials, whose primary outcome was the incidence of predefined adverse respiratory events, failed to show that a propofol–ketamine combination was superior to propofol [21,23,24]. However, two meta-analyses in 2015 and 2016 concluded that sedation using a propofol–ketamine combination had a lower incidence of adverse respiratory events [8,11]. A more recent multicenter randomized control trial [22], which was not included in the previous meta-analyses, also failed to show that a propofol–ketamine combination was superior to propofol. In our study, about half of the patients in group K2 appeared apneic. In most of these patients, apnea was likely due to upper airway obstruction, as it resolved after jaw thrust maneuver. Although previous studies also showed that some patients required airway interventions [21,22,23,24], the high rate of interventions in group K2 was exceptional. This high rate may be due to the distinct dosing regimen used in this study. All patients initially received a fixed dose of propofol (1 mg/kg), with those in groups K1 and K2 also receiving 0.33 mg/kg and 0.66 mg/kg ketamine, respectively. Thus, the total amount of sedative was highest in group K2. Other studies allowed flexibility in the total dosage of sedatives, reducing the inequality of sedatives among patient groups [21,22,23,24]. Sedation may have been deeper in group K2 than in the other groups, resulting in the lowest incidence of procedural interference and the highest rate of apnea in group K2. However, this study was unable to assess possible gaps in the sedation level, as the RSS cannot distinguish between moderate and deep sedation. Differences may be detected using other tools, such as modified RSS, Richmond Agitation–Sedation Scale (RASS), or modified Observer’s Assessment of Alertness/Sedation (OAA/S) Scale, which use painful stimuli to assess deeper levels of sedation. 

Results from Group K2 deserves special mention. Despite the comparison of the incidence of adduction motion between Groups K1 and K2 showed no statistical significance, still, the regimen used in group K2 showed the greatest risk reduction. Besides, the only group that showed the absence of pain recall was group K2. However, the superiority of the regimen used in group K2 cannot be concluded in the current study.

This study had several limitations. First, the detailed threshold for respiratory intervention was not predetermined. The decision to intervene when apnea was observed was made by each attending clinician. However, any biases in the threshold were likely minimized by blinding. Second, the diagnosis of apnea was based solely upon capnography. It is a more prudent way of detecting apnea to observe various clinical signs in addition to capnography. However, we believe that the ambiguity or subjectivity in making clinical decisions could have been minimized by using capnography. Third, the procedural extent was not assessed quantitatively, which may affect the primary outcome. However, the fact that there were no significant differences in procedural duration and cervical pathology among groups may indirectly indicate the equivalency of the procedural extent. Fourth, procedural pain itself was not assessed directly. Validated behavioral pain scale for sedated patients such as Behavioral Pain Scale (BPS) or Critical-Care Pain Observation Tool (CPOT) would be more appropriate rather than assessing the memory of intraoperative pain. Fifth, glycopyrrolate, as an antisialagogue, was administered in all cases. Its use in group P was unnecessary, although it was inevitable for proper blinding. Additionally, the last limitation was the lack of detailed data concerning patient recovery profiles. The incidence of recovery agitation did not differ in the three patient groups. However, recovery reactions after ketamine administration have a wide spectrum of manifestations, from mild mood change to physical combativeness [4,26]. Although the clinical significance of relatively mild reactions remains, these reactions may not have been detected. 

Although safety is the highest priority, sedation is regarded as successful only when it prevents any procedural interference or discomfort. The regimen administered to group K1, consisting of 1 mg/kg propofol and 0.33 mg/kg ketamine, showed an ideal profile, with no increase in adverse respiratory events and reduced procedural interference compared with group P. The regimen administered to group K2, consisting of 1 mg/kg propofol and 0.66 mg/kg ketamine, can be considered when a deeper level of sedation is required and airway management seems feasible.

## 5. Conclusions

In conclusion, a propofol–ketamine combination was more effective than propofol alone in reducing procedural interference during LEEPs. However, increasing the dose of ketamine showed no additional benefit.

## Figures and Tables

**Table 1:** Patient characteristics and cervical pathologies.

	Group P	Group K1	Group K2	
	(*n* = 40)	(*n* = 40)	(*n* = 40)	*p*
ASA 1/2	12/28	10/30	9/31	0.738
Age (years)	44.0 [36.0; 50.5]	48.5 [36.0; 54.5]	38.0 [34.5; 56.0]	0.620
Height (cm)	159.1 ± 5.3	157.3 ± 5.8	159.2 ± 7.0	0.964
Weight (kg)	58.0 [54.9; 68.2]	58.2 [55.7; 64.0]	58.6 [53.6; 66.3]	0.695
BMI (kg/m^2^)	24.6 [21.4; 26.4]	23.8 [21.4; 25.5]	23.2 [20.6; 25.8]	0.515
Cervical cytology				0.782
ASC-US	1 (2.5)	3 (7.5)	1 (2.5)	
LSIL	3 (7.5)	1 (2.5)	2 (5.0)	
ASC-H	10 (25.0)	6 (15.0)	4 (10.0)	
HSIL AIS	24 (60.0) 0 (0.0)	26 (65.0) 1 (2.5)	28 (70.0) 0 (0.0)	
SCC	1 (2.5)	1 (2.5)	1 (2.5)	
Adenocarcinoma	0 (0.0)	0 (0.0)	1 (2.5)	
Unknown	1 (2.5)	2 (5.0)	3 (7.5)	
Final histology				0.810
Normal or cervicitis	1 (2.5)	2 (5.0)	0 (0.0)	
CIN 1	4 (10.0)	3 (7.5)	2 (5.0)	
CIN 2	11 (27.5)	10 (25.0)	14 (35.0)	
CIN 3	19 (47.5)	18 (45.0)	19 (47.5)	
CIS AIS	1 (2.5) 0 (0.0)	0 (0.0) 2 (5.0)	1 (2.5) 0 (0.0)	
ASC-US	3 (7.5)	2 (5.0)	1 (2.5)	
ASC-H	0 (0.0)	1 (2.5)	0 (0.0)	
Adenocarcinoma	0 (0.0)	0 (0.0)	2 (5.0)	
SCC	1 (2.5)	2 (5.0)	1 (2.5)	

Data are presented as the number (%); median [interquartile range]; or as the mean ± standard deviation (SD). ASA, American Society of Anesthesiologists; BMI, body mass index; ASC-US, atypical squamous cells of undetermined significance; LSIL, low-grade squamous intraepithelial lesions; ASC-H, atypical squamous cells, cannotexclude HSIL; HSIL, high-grade squamous intraepithelial lesions; AIS, adenocarcinoma in situ; SCC, squamous cell carcinoma; CIN, cervical intraepithelial neoplasia; CIS, carcinoma in situ.

**Table 2:** Incidence of adduction motion as primary outcome.

	Group P	Group K1	Group K2	*p*	P vs. K1	P vs. K2	K1 vs. K2
Outcome					RR (95% CI)	RR (95% CI)	RR (95% CI)
					*p*	*p*	*p*
Adduction	27 (67.5%)	13 (32.5%)	4 (10%)	<0.001	2.08 (1.27 ~ 3.41)	6.75 (2.60 ~ 17.53)	3.25 (1.16 ~ 9.12)
No movement	13 (32.5%)	27 (67.5%)	36 (90.0%)		0.004	< 0.001	0.029

Data are presented as the number (%). RR, relative risk; CI, confidence interval. *P-*values were calculated using χ2 test.

**Table 3:** Intraoperative and postoperative results as secondary outcomes.

Outcome	Group P	Group K1	Group K2	*p*	P vs. K1	P vs. K2	K1 vs. K2
					*p*	*p*	*p*
Jaw thrust maneuver	8 (20.0)	4 (10.0)	17 (42.5)	0.002	0.348	0.054	0.002
Bag-mask ventilation	6 (15.0)	2 (5.0)	2 (5.0)	0.215			
Additional propofol	39 (97.5)	23 (57.5)	12 (30.0)	< 0.001	< 0.001	< 0.001	0.024
1	7 (17.5)	10 (25.0)	4 (10.0)				
2	32 (80.0)	13 (32.5)	8 (20.0)				
Sevoflurane inhalation	6 (15.0)	2 (5.0)	1 (2.5)	0.144			
Pain recall	3 (7.5)	3 (7.5)	0 (0.0)	0.244			
Recovery agitation	3 (7.5)	2 (5.0)	0 (0.0)	0.368			
Patient’s satisfaction	5 [4.5; 5.0]	5 [4.0; 5.0]	5 [5.5; 5.0]	0.322			
Surgeon’s satisfaction	3 [2.0; 4.5]	4 [4.0; 5.0]	5 [4.0; 5.0]	< 0.001	0.001	< 0.001	0.403
Procedure duration (sec)	495 [395.0; 595.0]	440 [384.0;519.0]	422 [390.0;485.0]	0.196			
PACU stay time (min)	30.3 ± 12.9	28.6 ± 11.7	27.8 ± 11.8	0.357			

Data are presented as the number (%); mean ± standard deviation; or median [interquartile range]. PACU, post-anesthesia care unit.
